# Supplementary figures and images for: Ternary regulation mechanism of Rhizoma drynariae total flavonoids on induced membrane formation and bone remodeling in Masquelet technique
Source: PLoS One. 2022 Dec 6;17(12):e0278688. doi: 10.1371/journal.pone.0278688 (PMC9725127; doi:10.1371/journal.pone.0278688)

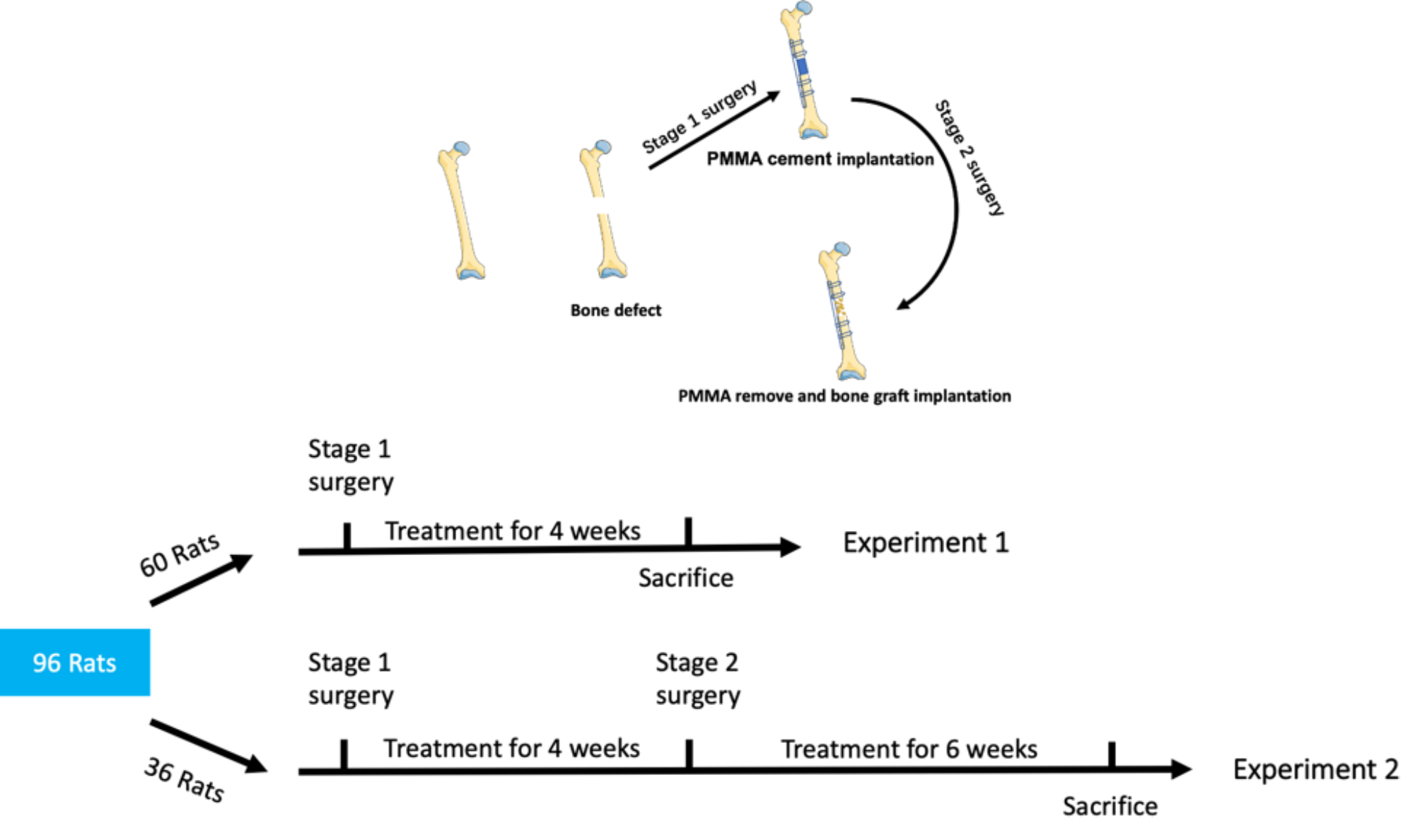

Supplement: S1 Fig — (JPG) [file pone.0278688.s001.jpg]

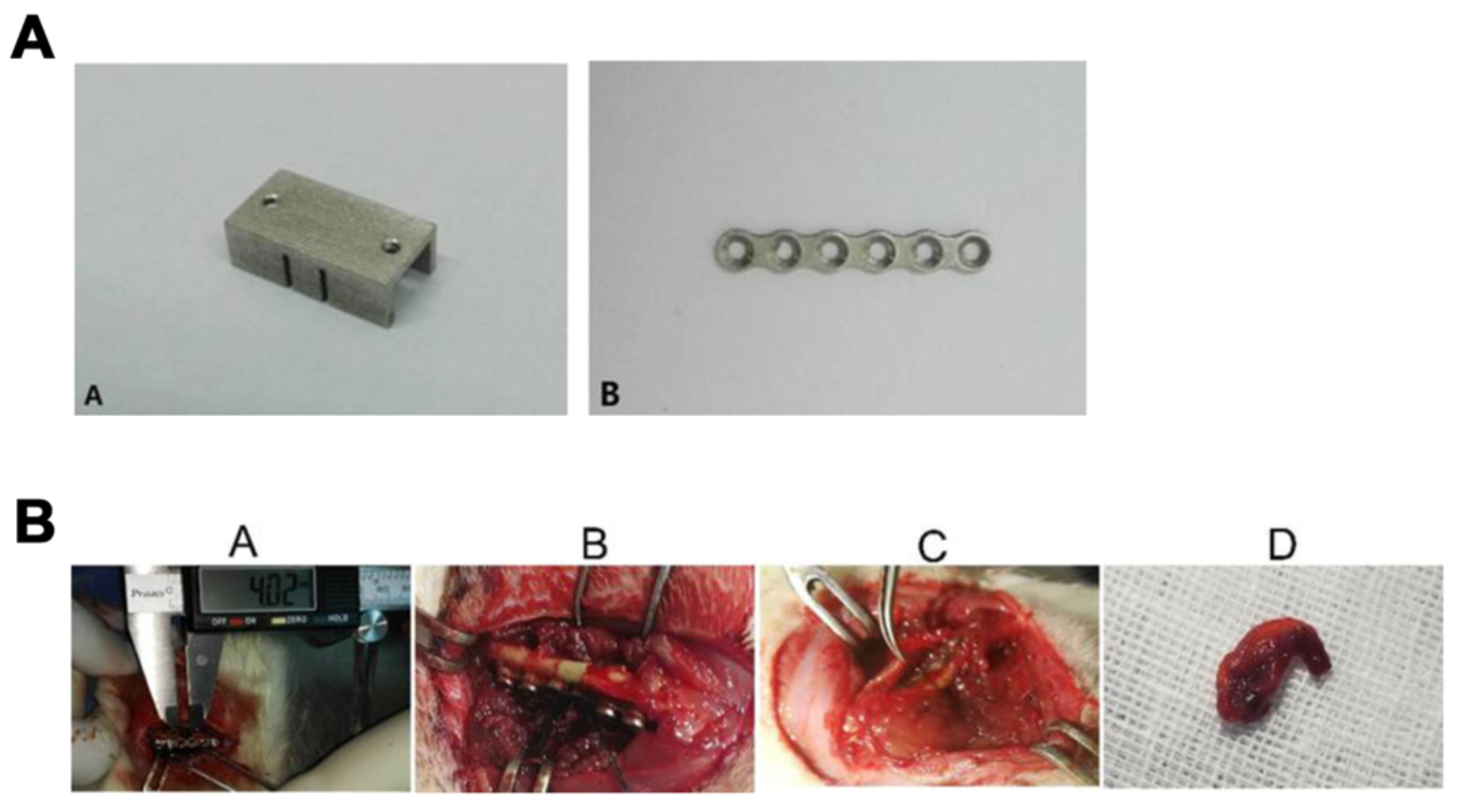

Supplement: S2 Fig — (A) Self-made femoral plate and osteotomy guide plate for rats. (B) The 4-mm precision osteotomy was performed using the osteotomy guide plate to create a rat femoral defect model (panel A). The bone defect area was filled with PMMA cement (panel B). Four weeks after the primary operation, the induction of the bone membrane was observed (panel C). The induced membrane tissue was taken out (panel D). (JPG) [file pone.0278688.s002.jpg]
